# Dissymmetry enhancement in enantioselective synthesis of helical polydiacetylene by application of superchiral light

**DOI:** 10.1038/s41467-018-07533-y

**Published:** 2018-11-30

**Authors:** Chenlu He, Guang Yang, Yan Kuai, Sizhen Shan, Lin Yang, Jingang Hu, Douguo Zhang, Qijin Zhang, Gang Zou

**Affiliations:** 1 CAS Key Laboratory of Soft Matter Chemistry, Department of Polymer Science and Engineering, iChEM, University of Science and Technology of China, 230026 Hefei, Anhui China; 2 College of Science, Northeast Forestry University, 150040 Harbin, China; 3 Department of Optics and Optical Engineering, Institute of Photonics, University of Science and Technology of China, Number 96 Jinzhai Road, 230026 Hefei, Anhui China

**Keywords:** Photocatalysis, Polymer characterization, Polymerization mechanisms

## Abstract

Superchiral light, generated by the interference of two counter-propagating circularly polarized light (CPL) with same frequency, opposite handedness and different intensity, exhibits enhanced dissymmetry in its interaction with chiral molecules, and has the potential for ultrasensitive detection and characterization of chiral molecules. It is anticipated that the enhanced optical dissymmetry in superchiral light (SCL) field may be utilized to promote asymmetric photochemical reactions efficiency. Herein we reported SCL impart greater chiral bias to trigger asymmetric photo-polymerization reaction from initially achiral diacetylene (DA) monomer, and the enhanced optical dissymmetry for whole polydiacetylene (PDA) films could be achieved. An explanation based on the chiral transfer and amplification of chiral bias from SCL during the polymerization process has been proposed. Moreover, thus formed chiral PDA films polymerized by SCL exhibited enhanced enantioselective recognition ability, and can serve as a direct visual probe for the discrimination of some specific enantiomers.

## Abstract (web summary)

Superchiral light can be utilized to promote asymmetric photochemical reactions. Here the authors show that superchiral light imparts greater chiral bias to trigger asymmetric photo-polymerization reactions from initially achiral diacetylene monomers and enhanced optical dissymmetry in polydiacetylene films.

## Introduction

Circularly polarized light (CPL) has attracted considerable interests since it is a possible explanation for the origin of the homochirality in nature^1–3^. Recently, CPL has been widely applied in asymmetric photochemistry because it could impart initial chiral bias into chemical reactions^4–6^. By using left- and right-handed CPL, people have successfully achieved asymmetric photolysis as well as photosynthesis of amino acid derivatives^7^, asymmetric polymerization for PDA and chiral coordination polymers^8^, helical structure modulation in azobenzene-containing polymers^9^, polyfluorene^10^, and so on. However, in most above cases, only a small enantiomeric excess (<4%) can be obtained since their anisotropy (g) factors were very small (<10^−3^)^11,12^. In this sense, there is good motivation to find generic mechanisms to further enhance dissymmetry in CPL-triggered enantioselective reactions, which may open the door to promote absolute asymmetric synthesis in which light provides the chiral bias.

Superchiral light (SCL) field, which can be easily generated by the optical excitation of plasmonic planar chiral metematerials^13^ or by the interference of two counter-propagating CPL beams^14^, is assumed to display greater chiral dissymmetry than CPL, allowing ultrasensitive detection of chiral molecules^15^. However, in SCL field, the enhanced optical dissymmetry is restricted to very narrow regions (nearby the chiral surface plasmons or near the nodes of generated SCL field)^16^, limiting their practical applications. To the best of our knowledge, attempts to trigger asymmetric photochemical reactions by application of SCL have rarely been described to date. Moreover, as reported previously, cooperative response to small chiral bias and amplification effect occurred during the polymerization would be extremely large^17^. In this sense, owing to the chiral transfer and amplification during polymerization process, SCL might be utilized to introduce greater chiral bias into photo-polymerizations although the enhanced optical dissymmetry in SCL field is usually restricted to very narrow regions. Herein, we demonstrated experimentally that the enantioselective polymerization of achiral diacetylene monomer can be greatly enhanced by application of SCL, and a maximum about 6-fold enhancement over CPL can be achieved. The enhancement in enantioselective synthesis of helical PDA chains can be rigorously controlled with the relative light intensity ratio of the two counter-propagating CPL waves. This work not only provides a methodology for promoting the light-triggered absolute asymmetric synthesis, but also is of great basic value for further understanding of symmetry breaking in asymmetric photo-photochemical reactions and the origins of biomolecular homochirality in living matter.

## Results

### Synthesis of chiral PDA films with SCL

**Fig. 1:**
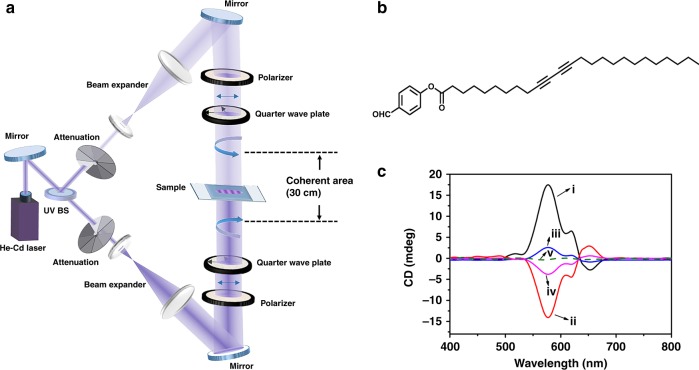
Experimental set-up, molecular structure and CD spectra. **a** Experimental set-up for SCL generated by two counter-propagating CPL waves with same frequency and opposite handedness. The coherent length of the laser is 30 cm and the optical path difference of the two counter-propagating CPL waves is less than 2 cm. **b** The molecular structure of BSDA monomer. **c** CD spectra of thus-formed PDA films by application of (i) left-handed or (ii) right-handed SCL; (iii) left-handed or (iv) right-handed CPL; (v) LPL, respectively. The wavelength of SCL, CPL and LPL were all 325 nm. The irradiation time was 40 min

Asymmetric photo-polymerization reactions of achiral benzaldehyde-functionalized diacetylene (BSDA) monomer were carried out by application of SCL generated by the interference of two counter-propagating CPL beams (325 nm, same frequency, opposite handedness, 4.0 and 2.8 mW cm^−2^, respectively), as shown in Fig. 1a. The spatial period of the generated SCL field was 162.5 nm, as illustrated in Supplementary Fig. 1 (see Methods for details). Since the optical dissymmetry was enhanced greatly near the nodes of the generated SCL field^18^, 61-layers BSDA LB films were fabricated with an estimated thickness of about 164 nm, to ensure that there was at least one node of the SCL field located within the photo-polymerization region (see Methods and Supplementary Fig. 2 and Fig. 3 for details). Photo-polymerization reactions of achiral BSDA monomer (Fig. 1b) were performed by using SCL, CPL or linearly polarized light (LPL), respectively. After photo-polymerization, PDA films exhibited blue phase and typical intense absorption maximum at about 647 and 596 nm, indicating the successful polymerization of BSDA monomer (fractional conversion was nearly 0.21) and formation of PDA chains (Supplementary Fig. 4). When above films were subjected to the characterization of circular dichroism (CD), it was interesting to note that the samples polymerized with SCL exhibited stronger CD signals than those polymerized with conventional CPL, while the samples polymerized with LPL exhibited CD silence. To exclude possible effect of linear dichroism of the films, CD characterizations were performed by rotating the sample about the normal of the films, and the signal intensity hardly changed with the rotation angle, indicating that the main origin of CD signals should be ascribed to the helical conformation of PDA chains (Supplementary Fig. 5). Upon heating all samples to 70 °C for 10 min to confirm the stability of CD signals, all of them turned red from blue phase and exhibited typical absorption maximum at about 532 and 485 nm owing to the classical thermochromic phase transition of PDA backbone^19^. It is interesting to note here that CD signals of above samples maintained, but showed a corresponding blue shift as that of the ultraviolet (UV) absorption band during annealing process (Supplementary Fig. 6).

**Fig. 2:**
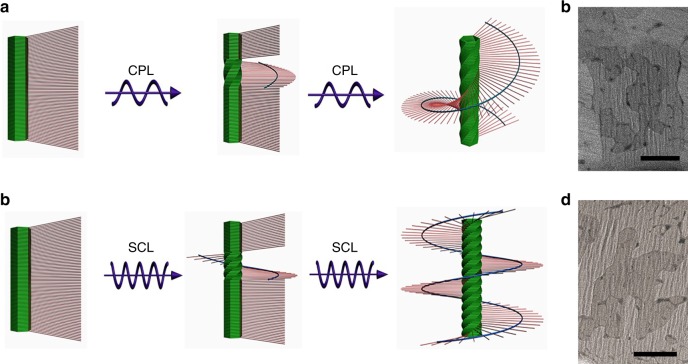
Possible helix formation mechanism and TEM characterization. Scheme for asymmetric photo-polymerization mechanism for helical PDA chains prepared by application of **a** CPL and **c** SCL irradiation. TEM imagines of helical PDA chains prepared with **b** CPL and **d** SCL. TEM imagines revealed that more helical PDA chains were formed by application of SCL than polymerized with CPL. The arrows in **a** and **c** represented CPL and SCL, respectively. Scale bars, 200 nm

To quantitatively evaluate the dissymmetry enhancement effect, we defined the absorption dissymmetry factor (*g*) as *g* = (*ε*_L_ − *ε*_R_)/[(*ε*_L_ + *ε*_R_)/2], where |*g*| < 2. The *ε*_L_ and *ε*_R_ were defined as the absorption coefficient of left-handed and right-handed polarized light, respectively^20^. As shown in Supplementary Fig. 7, the samples polymerized with left-handed or right-handed SCL clearly exhibited opposite *g* factor at the corresponding absorption band for PDA chains, and *g* values at 577 nm were 2.62 × 10^−4^ and −2.53 × 10^−4^, respectively. However, in the case of CPL irradiation, the samples exhibited *g* values of 0.48 × 10^−4^ and −0.50 × 10^−4^, respectively. Nearly 5-fold enhancement in g values for whole PDA films could be achieved, which should be ascribed to the enhanced optical dissymmetry in SCL field. SCL field appeared to have stronger twist optical forces near the nodes and might impart greater chiral bias into asymmetric photo-polymerization reactions. Although the enhanced optical dissymmetry in SCL field was always restricted to narrow region (only near the nodes), the greater enhancement in *g* values for whole PDA films could be achieved. In LB films, BSDA monomers were arranged closely and orderly, with strong synergistic effect and sensitive cooperative response to the chiral bias. Therefore, compared with well-established techniques using CPL, the enhanced optical dissymmetry in SCL within very narrow region would be transferred and amplified during asymmetric photo-polymerization process^17^, eventually resulted in the formation of more helical PDA chains predominantly in one chiral state, as well as the greater enhancement in g values for whole PDA films, as illustrated in Fig. 2. Transmission electron microscopy (TEM) characterization also revealed that more helical PDA chains were formed by application of SCL than those polymerized with CPL alone (Fig. 2b–d). Moreover, the induced chirality excess (DCE) values for the samples polymerized by using left-handed SCL and CPL were calculated to be 0.25 ± 0.04 and 0.06 ± 0.03, respectively, through second-harmonic generation (SHG) combined with linear dichroism (SHG-LD) method (Supplementary Fig. 8)^21^. Nearly four-fold enhancement in DCE values for the PDA films polymerized by application of SCL over CPL could be observed, indicating that SCL could impart greater chiral bias into asymmetric photo-polymerization reactions, eventually promoted the enantioselective synthesis of helical PDA chains.

### Asymmetric photo-polymerization kinetics

**Fig. 3:**
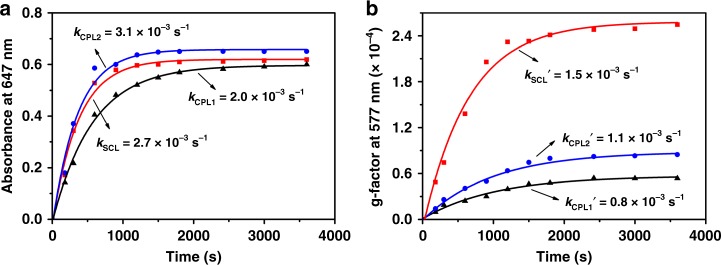
Photo-polymerization kinetics. **a** Time-resolved development of absorption maximum at 647 nm for PDA chains upon irradiation with SCL (red lines), CPL1 (4.0 mW cm^−2^, black lines) and CPL2 (6.8 mW cm^−2^, blue lines). **b** Time-resolved development of g values maximum at 577 nm for helical PDA chains predominantly in one chiral state upon irradiation with SCL, CPL1, and CPL2. Solid curves in **a** and **b** are fitted with single exponential function

To further prove the hypothesis, asymmetric polymerization experiments were carried out upon irradiation with SCL or CPL (4.0 or 6.8 mW cm^−2^), respectively. The polymerization kinetics could be investigated using the chronological development of the absorption maxima at 647 nm for the formation of PDA chains. The photo-polymerization of BSDA monomer could be analyzed as ln[(*A*_∞_ − *A*_t_)/(*A*_∞_ − *A*_0_)] = −*kt*, where *A*_0_, *A*_t_, and *A*_∞_ are the absorbance (647 nm) at the initial time, time *t* and the photo-stationary state, *k* is the photo-polymerization rate constant and t is the irradiation time^22^, respectively. As shown in Fig. 3a and Supplementary Fig. 9a, all polymerization processes could be regarded as first order reaction, and the polymerization rate constant for SCL or CPL irradiation were estimated to be 2.7 × 10^−3^ s^−1^ (*k*_SCL_), 2.0 × 10^−3^ s^−1^ (*k*_CPL1_, 4.0 mW cm^−2^) or 3.1 × 10^−3^ s^−1^ (*k*_CPL2_, 6.8 mW cm^−2^), respectively. Obviously, the polymerization kinetic upon SCL irradiation was slightly slower than that upon CPL irradiation (6.8 mW cm^−2^), which should be ascribed to the effect of the light intensity. In addition, it was reasonable to use the chronological development of the g-factor extremum at 577 nm to evaluate the asymmetric polymerization kinetics. The asymmetric photo-polymerization of BSDA monomer could also be analyzed as ln[(*g*_∞_ − *g*_t_)/(*g*_∞_−*g*_0_)] = −*k*′*t*, where *g*_0_, *g*_t_, and *g*_∞_ are the *g*-values (577 nm) at the initial time, time *t* and the photo-stationary state, and *k*′ is the asymmetric photo-polymerization rate constant, respectively. As shown in Supplementary Fig. 10, the g-factor extremum at 577 nm increased with the increasing of irradiation time, which should be ascribed to the formation of helical PDA chains in blue phase. Meanwhile, the kinetic curves also proved that the asymmetric polymerization process could be regarded as a first order reaction (Fig. 3b, Supplementary Fig. 9b and Table 1), the asymmetric polymerization rate constant, *k*_SCL_′, *k*_CPL1_′ (4.0 mW cm^−2^) and k_CPL2_′ (6.8 mW cm^−2^) were estimated to be 1.5 × 10^−3^ s^−1^, 0.8 × 10^−3^ s^−1^, and 1.1 × 10^−3^ s^−1^, respectively. It is interesting to note here that the formation of helical PDA chains predominantly in one chiral state proceed more fast in the case of SCL irradiation, although the polymerization kinetic upon SCL irradiation was slightly slower than that upon CPL irradiation (6.8 mW cm^−2^). All above results confirmed that SCL appeared to impart greater chiral bias over CPL in triggering asymmetric photo-polymerization reactions, favoring for producing more helical PDA oligomers predominantly in one chiral state. The helical oligomer radical could react more easily with the neighbor monomer, eventually resulted in the formation of PDA film with predominantly one helical sense.

### Asymmetric photo-polymerization mechanism

**Fig. 4:**
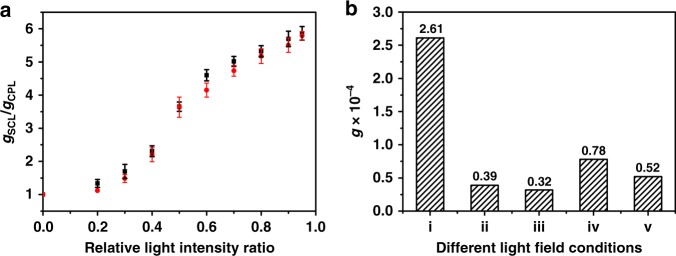
Dissymmetry enhancement mechanism. **a** The relative enhancement factor, *g*_CSL_/*g*_CPL_ for final PDA films as a function of the relative light intensity ratio, *R*, by application of left-handed (black dots) or right-handed SCL(red dots). At least four irradiation experiments were performed to obtain the average *g*-value, and error bars represented standard deviations of measured *g* values. **b** The *g* value at 577 nm for final PDA films upon irradiation with two counter-propagating CPL beams with (i) opposite or (ii) same handedness; two CPL beams propagated along the same direction with (iii) opposite or (iv) same handedness; or (v) one CPL beam (4.0 mW cm^−2^), respectively

In order to distinguish the enhancement mechanism, the relative enhancement factor, *g*_SCL_/*g*_CPL_, for the final PDA films was explored as a function of the relative light intensity ratio, *R* of the two counter-propagating CPL beams. The detail data for above different light intensities were shown in Supplementary Table 2. It was apparent that the absolute *g* values of final PDA films increased with the increasing of *R*, as illustrated in Fig. 4a. When *R* was less than 0.4, no obvious enhancement in *g* values (*g*_SCL_/*g*_CPL_ < 2), could be detected for final PDA films, which should be ascribed to the weak interference effect of the two counter-propagating CPL^23^. In the region of 0.4 < *R* < 0.6, the *g*_SCL_/*g*_CPL_ increased rapidly with the increasing of *R*, which could be ascribed to the dramatically enhanced optical dissymmetry near the nodes of the SCL field, which could be predicted by equation *g*_SCL_/*g*_CPL_ = (1+$\sqrt R$)/(1‒$\sqrt R$)^14^. In the region of 0.7 < *R* < 1.0, the obtained *g*_SCL_/*g*_CPL_ values for final PDA films gradually saturated, gaining its maximum value of 5.8. We must note that the dissymmetry enhancement in above asymmetric photo-polymerization depended on not only the greater chiral bias induced by SCL, but also the chiral transfer and amplification efficiencies during the polymerization process, which might strongly depended on the closely and orderly packing of BSDA monomers in LB films^17^. In the region of 0.7 < *R* < 1.0, the enhanced optical dissymmetry in SCL field increased with the increasing of *R*, however, the packing of BSDA monomers in LB films were predetermined, limiting further greatly promotion of asymmetric photo-polymerization and the enhancement in *g* value for whole PDA films^24,25^.

Moreover, in order to further distinguish the effect of the enhanced optical dissymmetry in SCL field, serials of control experiments were carried out by varying the propagating direction or the handedness of the two CPL beams. As shown in Fig. 4b and Supplementary Fig. 11, upon irradiation with two counter-propagating CPL beams but same handedness, the optical dissymmetry of the standing wave field would be greatly weakened^18^. So the *g* values of PDA films was only 0.39 × 10^−4^ in this case, even lower than that upon irradiation with one CPL beam (0.52 × 10^−4^, 4.0 mW cm^−2^). If two CPL beams propagated along the same direction with fixed phase difference, they would interfere with each other, but couldn’t generate SCL field. The *g* values of final PDA films were only 0.78 × 10^−4^ (with same handedness) and 0.32 × 10^−4^ (with opposite handedness), respectively, much lower than that upon irradiation with two counter-propagating CPL beams with opposite handedness (*g* value = 2.62 × 10^−4^). Based on above discussion, the mechanism of enhanced asymmetric photo-polymerization was directly owing to the enhanced optical dissymmetry in SCL field generated by the interference of two counter-propagating CPL beams with same frequency but opposite handedness, instead of the interference effect itself.

### Discrimination of specific enantiomers

**Fig. 5:**
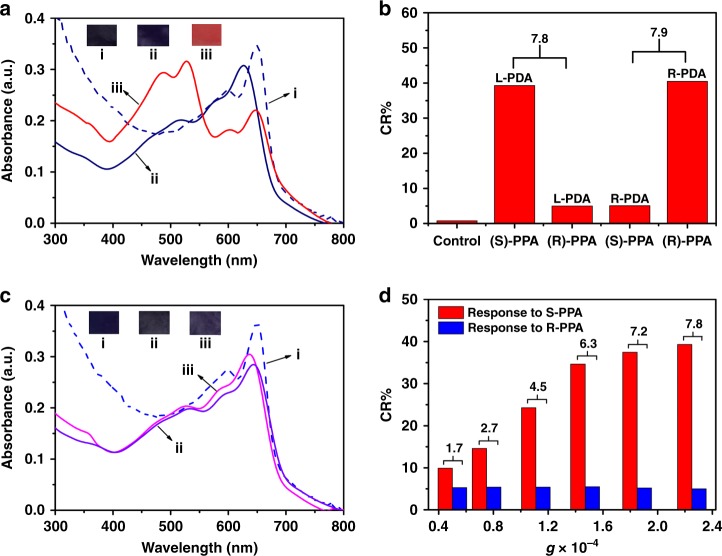
UV–vis spectra and CR values. UV–vis absorption spectra of chiral PDA films prepared with left-handed **a** SCL and **c** CPL: (i) before treatment, or upon immersing into (ii) R-type or (iii) S-type PPA solution for 10 min. The insert exhibits corresponding color changes. **b** The CR values of chiral PDA films prepared with SCL on response to R-type or S-type PPA solution. **d** The CR values of chiral PDA films prepared with left-handed SCL with various *g* values on response to S-type and R-type PPA enantiomers

Recently a simple protocol for enantioselective discrimination of enantiomers is extremely intriguing since enantiomers generally exhibited different biological activities, especially in biomedical and pharmaceutical analysis^26^. As reported previously, chiral PDA films prepared with CPL technique could be applied for the direct visual enantioselective recognition of amino acids enantiomers^27^. However, the efficiency of CPL-triggered asymmetric photo-polymerization was still very low, limiting the practical application of thus-formed chiral PDA materials. It is anticipated that the optical dissymmetry of chiral PDA films could be enhanced by using SCL instead of CPL, eventually resulted in the enhancement of their enantioselective discrimination properties. As reported previously, the optically active 1-phenylpropanol were important as synthetic intermediates of various functionalities such as halide, amine, ester, ether, and so on^28^. Therefore, the discrimination properties of thus-formed PDA films prepared by CPL or SCL were investigated by UV–Vis absorption characterization in detail upon immersing above films into 1-phenylpropanol (PPA, Supplementary Fig. 12) enantiomers aqueous solution (0.01 M) for 10 min. As shown in Fig. 5a, obvious blue-to-red color transition was observed for chiral PDA films prepared by left-handed SCL upon immersing into S-type PPA solution, while weak color change could be detected for samples upon immersing into R-type PPA solution. As for the samples prepared with left-handed CPL, the change of color was less obvious upon immersing into R-type or S-type PPA solution. Namely, chiral PDA films prepared with SCL could be applied for the direct visual discrimination of PPA enantiomers with greater enantioselective discrimination ability, while hard for the films prepared with traditional CPL techniques. To further evaluate the enantioselective discrimination properties of chiral PDA films, we defined the colorimetric response (CR) as the relative change in percent of blue phase, defined as CR = [PB_0_ − PB_1_]/PB_0_ × 100%, where PB_0_, PB_1_ was the initial and final percent of blue phase in PDA films before and after immersion into PPA enantiomers solution, respectively. As shown in Fig. 5b, chiral PDA films prepared with left-handed SCL exhibited high CR value (39%) upon response to S-type PPA, while low CR value (5%) upon to R-type PPA. Interestingly, opposite enantioselective discrimination behavior could be obtained for the samples prepared with right-handed SCL, which exhibited high CR value (40%) upon response to R-type PPA, while low CR value (5%) upon to S-type PPA. However, as for the samples prepared by traditional CPL technique, only low CR values (10% for S-type, and 6% for R-type PPA enantiomers, respectively) could be detected, owing to their weaker enantioselective discrimination ability (Supplementary Fig. 13).

It was reasonable to use the chronological development of the CR value to study the solvatochromatic transition kinetics of chiral PDA films upon immersion into PPA enantiomers solution. As shown in Supplementary Fig. 14, upon immersing into S-type PPA enantiomers solution, PDA films prepared with left-handed SCL quickly turned red and its CR value reached approximately 30% within 6 min. While immersing into R-type PPA enantiomers solution, their solvatochromatic transition became relatively slowly, and its CR value gained maximum (5%) over 10 min. Their high enantioselective discrimination properties should be attributed to an inherent chiral recognition based on the steric repulsion of helical PDA assemblies towards the opposite chiral PPA molecules^29^. As for the samples prepared with CPL, only weaker enantioselective discrimination behavior could be detected. All above results confirmed that chiral PDA films prepared by SCL exhibited stronger enantioselective discrimination ability for PPA enantiomers than those prepared by conventional CPL, which should be ascribed to the enhanced optical dissymmetry of final PDA films prepared by SCL. In order to further confirm the above-mentioned hypothesis, chiral PDA films with various *g* values were prepared with SCL by varying the relative light intensity ratio of the two counter-propagating CPL beams and their enantioselective discrimination properties to PPA enantiomers were investigated in detail. It was apparent that their enantioselective discrimination ability increased with the increasing of the *g* values of chiral PDA films (Fig. 5d). It is anticipated that the dissymmetry in asymmetrical photo-polymerization would be further enhanced by varying the distribution of the chiral light filed (e.g., the combination of the far-field and near-field optics), which might pave the way for amplification of a small chiral bias toward absolute asymmetric synthesis. Inspired by the preliminary results, we further investigated the enantioselective discrimination ability of the prepared helical PDA assemblies toward other chiral compounds, including 1-phenylethanol (PEA), 1-phenylethylamine (PEAM) and 1-phenylpropylamine (PPAM) enantiomers (Supplementary Fig. 12). Clearly, the samples polymerized with SCL exhibited better enantioselective discrimination ability toward to PEA enantiomers (Supplementary Fig. 15) similar as that for PPA, while weak discrimination ability toward PEAM or PPAM enantiomers (Supplementary Fig. 16 and 17). The interactions between the aromatic aldehyde and the amino groups was too strong, unfavorable for excellent enantioselective discrimination ability. Therefore, suitable interaction between aromatic aldehyde and OH groups was assumed to play a key role in excellent enantioselective discrimination ability of helical PDA assemblies.

## Discussion

CPL is considered to be a true chiral entity and can induce absolute asymmetric synthesis, however, the enantiomeric excess in CPL-triggered asymmetric photo-chemical reactions are extremely small. In this sense, an amplification mechanism is absolute essential to enhance the initial chiral bias to induce absolute asymmetric synthesis. SCL, appeared to display greater enantioselective interaction with chiral molecules than conventional CPL, has been utilized as a strategy to impart greater chiral bias into photo-chemical reactions. In addition, based on the chiral transfer and amplification during polymerization process, the small chiral perturbation induced by SCL could be further amplified and had been utilized to synthesis macroscopic chiral PDA films with enhanced optical activity and enantioselective discrimination ability. This work not only provides a methodology for promoting light-triggered absolute asymmetric synthesis, but also is of great fundamental value for a deeper understanding of the amplification of chiral bias in asymmetric photo-photochemical reactions and the origin of homochirality in nature.

In summary, the present study experimentally demonstrates that symmetry breaking in photo-polymerization from initially achiral DA monomer could be further enhanced by application of SCL field, generated by the interference of two counter-propagating CPL beams. Although the enhanced optical dissymmetry in SCL field is always restricted to narrow regions near the nodes of the SCL field, our results showed convincingly that the apparent enhancement in *g* values for whole PDA films could be achieved, and an explanation based on the chiral transfer and amplification during photo-polymerizations process had been proposed. Thus-formed PDA films prepared with SCL exhibited enhanced enantioselective discrimination ability than those prepared by conventional CPL technique, and could serve as a direct visual probe for the discrimination of PPA enantiomers. We hope these results would be expanded to other asymmetric photochemical or polymerization systems, which might open pathway for promoting light-triggered absolute asymmetric synthesis.

## Methods

### Materials

10, 12-Pentacosadiynoic acid (PCDA) was purchased from Tokyo Chemical Industry Co., Ltd., and purified by dissolving in cyclopentanone and subsequently filtrating to remove polymer before used. A benzaldehyde-substitute diacetylene (BSDA) were synthesized in analogy to the previous procedure^30^. The photoresist, AR3120, was purchased from ALLRESIST, Germany, and used as received. All other solvents and reagents were of analytical grade and used as received. Milli-Q water (18.2 MΩ cm) was used in all cases. A KSV Mini-trough (KSV Instruments Ltd, Finland) was employed for depositing multilayer BSDA Langmuir–Blodgett (LB) films. The compound was dissolved in chloroform with a concentration of 1.0 mg mL^−1^. After waiting 30 min for the solvent evaporation, the monolayer could be easily transferred onto the quartz slice at a surface pressure of 30 mN m^−1^ with the transfer ratio near 1.0 by a vertical lifting technique (the lifting ration was about 5 mm min^−1^). Nearly 164 nm LB films containing 61 layers of BSDA monomer were prepared.

### Fabrication of superchiral light field

SCL could be generated by the interference of two counter-propagating CPL beams with same frequency, opposite handedness, slightly different intensity and a fixed phase difference. The detailed experimental setup for the generation of SCL was shown in Fig. 1a. A beam splitter was used to divide the incident laser beam (325 nm from 7511-G He-Cd Laser) into two beams, which were used to generate one left-handed and another right-handed CPL beams, respectively, with the combination of a linear polarizer and a quarter-wave plate. The two CPL beams has the same frequency, opposite handedness, a fixed phase difference, and the intensity of each CPL beam could be easily adjusted from 0 to 4 mW cm^−2^ by an adjustable attenuator. The coherent length of the laser is 30 cm and the optical path difference between the two CPL beams was less than 2 cm, allowing the interference of above two CPL beams. One CPL (4.0 mW cm^−2^) beam was directly passed through the sample films at normal incidence, and another counter-propagating CPL beam (same frequency, opposite handedness, 2.8 mW cm^−2^) would interfere with the incident CPL beam in order to generate SCL field. In addition, the diameter of the light spot was about 1 cm, located in the center of films.

As we all know, CPL can be decomposed into two linearly polarized light with orthogonal polarization direction and a phase deviation of ${\textstyle{\pi \over 2}}$. The left-handed or right-handed CPL propagating in the *Z* axis direction can be expressed as:

1 $$\begin{array}{l}\tilde E_{\mathrm{L}} = \tilde e_x{{A}}_{\mathrm{L}}{\mathrm{cos}}\left( {kz - \omega t} \right) + \tilde e_y{{A}}_{\mathrm{L}}{\mathrm{cos}}\left( {kz - \omega t + \frac{\pi }{2}} \right)\\ \hskip 1.9pc = \tilde e_x{{A}}_{\mathrm{L}}{\mathrm{cos}}\left( {\frac{{2\pi }}{\lambda }z - \omega t} \right) + \tilde e_y{{A}}_{\mathrm{L}}{\mathrm{cos}}\left( {\frac{{2\pi }}{\lambda }z - \omega t + \frac{\pi }{2}} \right)\end{array},$$

2 $$\begin{array}{l}\tilde E_{\mathrm{R}} = \tilde e_x{{A}}_{\mathrm{R}}{\mathrm{cos}}\left( {kz + \omega t} \right) + \tilde e_y{{A}}_{\mathrm{R}}{\mathrm{cos}}\left( {kz + \omega t - \frac{\pi }{2}} \right)\\ \hskip 1.8pc= \tilde e_x{{A}}_{\mathrm{L}}{\mathrm{cos}}\left( {\frac{{2\pi }}{\lambda }z + \omega t} \right) + \tilde e_y{{A}}_{\mathrm{L}}{\mathrm{cos}}\left( {\frac{{2\pi }}{\lambda }z + \omega t - \frac{\pi }{2}} \right)\end{array},$$

where $\tilde E_{\mathrm{L}}$ and $\tilde E_{\mathrm{R}}$ are the electric field vector of left-handed and right-handed CPL with amplitude of *A*_L_ and *A*_R_, $\tilde e_x$, and $\tilde e_y$ are the electric field unit vector in *x* and *y* direction, respectively. ${{k}} = {\textstyle{{2\pi } \over \lambda }}$, *ω* and *λ* are the wave vector, angular frequency and wavelength of above two counter-propagating CPL beams, respectively. The electric field vector of two counter-propagating CPL in the coherent area could be simply described:

3 $$\begin{array}{l}\tilde E_{\mathrm{L}} + \tilde E_{\mathrm{R}} = \tilde e_x\left[ {{{A}}_{\mathrm{L}}{\mathrm{cos}}\left( {kz - \omega t} \right) + {{A}}_{\mathrm{R}}{\mathrm{cos}}\left( {kz + \omega t} \right)} \right]\\ \hskip 2pc+ \tilde e_y\left[ {{{A}}_{\mathrm{L}}{\mathrm{cos}}\left( {kz - \omega t + \frac{\pi }{2}} \right) + {{A}}_{\mathrm{R}}{\mathrm{cos}}\left( {kz + \omega t - \frac{\pi }{2}} \right)} \right]\end{array},$$

there is only slight difference in *A*_L_ and *A*_R_ in the case of high interference contrast^14^, *A*_L_ and *A*_R_ could both be replaced with *A*. Therefore, above equation for SCL field could be simply expressed as:

4 $$\tilde E_{\mathrm{{SCL}}} = \tilde E_{\mathrm{L}} + \tilde E_{\mathrm{R}} = 2A{\mathrm{cos}}(kz)\left[ {\tilde e_x{\mathrm{cos}}(\omega t) + \tilde e_y{\mathrm{cos}}\left( {\omega t - \frac{\pi }{2}} \right)} \right],$$

in which the phase term $\tilde e_x{\mathrm{cos}}\left( {\omega t} \right) + \tilde e_y{\mathrm{cos}}\left( {\omega t - \frac{\pi }{2}} \right)$, implies that SCL in the whole coherent area is circularly polarized, while the amplitude term 2*A*cos(*kz*), describes the amplitude of SCL field, is similar to that of the generated standing wave field. The intensity of SCL, *I*_SCL_ can be obtained by modular squaring the amplitude $\tilde E_{\mathrm{{SCL}}}$ as:

5 $$I_{\mathrm{{SCL}}} \propto \left| {\tilde E_{\mathrm{{SCL}}}} \right|^2 = 4A^2{\mathrm{cos}}^2\left( {kz} \right) = 4A^2\left[ {\frac{{\cos \left( {2kz} \right) + 1}}{2}} \right] = 4A^2\left[ {\frac{{\cos \left( {\frac{{2\pi }}{{\lambda /2}}z} \right) + 1}}{2}} \right],$$

where ${\textstyle{{2\pi } \over {\lambda /2}}}$ in above equation implies that the spatial period of the generated SCL field should be *λ*/2. Based on above discussion, the spatial periods of the generated SCL field should be the same as the spatial period of the standing wave field (**∧**_SW_ = *λ*/2)^31^.

To confirm the formation of the SCL field, a 200 nm-thick photoresist (AR3120) film on glass slide was placed in above apparatus with a slight wedge angle of 5.5^o^ (Supplementary Fig. 1a) between the face of the glass slide and the plane perpendicular to the CPL beam. After development, a continuous photoresist stripes were formed on the glass slide with the spatial period of about 1.67 μm (**∧**_grating_, Supplementary Fig. 1b), indicating successfully formation of the SCL field by the interference of two counter-propagating CPL beams. Therefore, the spatial period of the generated SCL field along the CPL beam could be obtained as **∧**_SW_ =  **∧**_grating_ × sin(5.5^o^) = 160 nm, nearly in accordance with the theoretical calculated value, 162.5 nm (*λ*/2). Since the optical dis-symmetry was enhanced greatly near the nodes of the generated SCL field, 61-layers BSDA LB films were fabricated with an estimated thickness of about 164 nm (Supplementary Fig. 2), to ensure that there was at least one node of the generated SCL field within the whole film thickness. AFM characterizations revealed that the thickness of prepared BSDA LB films was about 166 nm (Supplementary Fig. 3), comparable to the estimated thickness and the spatial period of the generated SCL field. It is anticipated that the enhanced dissymmetry near the nodes of SCL field may impart greater chiral bias during the asymmetric photo-polymerization reaction, and eventually enhanced dissymmetry in enantioselective synthesis of helical PDA chains from initially achiral DA monomer could be achieved. As the reference samples, the photo-polymerization were carried out by application of CPL or LPL, and the light intensity were 4.0 mW cm^‒2^.

### Mechanism for the dissymmetry enhancement of SCL field

The dissymmetry factor in SCL field can be expressed as

6 $${{g}}_{{\mathrm{SCL}}} = {{g}}_{{\mathrm{CPL}}}\left( {\frac{{cC}}{{2U_{\mathrm{e}}\omega }}} \right),$$

where *g*_SCL_ and *g*_CPL_ are the dissymmetry factor under SCL and CPL, *c* is the speed of light, *C* is the optical chirality, *U*_e_ is the local electric energy density and *ω* is the angular frequency, respectively^14^. In CPL, the field vectors rotate at a constant rate along the propagation direction, undergoing a complete revolution once per wavelength, and the quantity ${\textstyle{{cC} \over {2U_{\mathrm{e}}\omega }}} = 1$. However, in the generated SCL field, $C = {\textstyle{{\omega _0(E_1^2 - E_2^2)} \over C}}$, independent of position. $U_{\mathrm{e}} = {\textstyle{\varepsilon_0 \over {2\left[ {E_1^2 + E_2^2 - 2{{E}}_1{{E}}_2{\mathrm{cos}}(2{{kz}})} \right]}}}$ and the minimum energy density appeared at the node as $U_{\mathrm{e}} = {\textstyle{{\varepsilon _0\left( {{{E}}_1 - {{E}}_2} \right)^2} \over 2}}$. Near a node of SCL field, *E*_2_ approaches *E*_1_, So *U*_e_ and *C* approach zero. But *U*_e_ approaches zero much faster than *C*, so the ratio ${\textstyle{{{C}} \over {{{U}}_{\mathrm{e}}}}}$ becomes extremely large, inducing *g*_SCL_**/***g*_CPL_ > 1.^18^ Since the relative light intensity ratio $R = \frac{I_{2}}{I_{1}} = \frac{E_{2}^{2}}{E_{1}^{2}}$, the dissymmetry factor near the nodes of SCL field can be expressed as *g*_SCL_ = *g*_CPL_ × ${\textstyle{{\left( {1 + \sqrt R } \right)} \over {\left( {1 - \sqrt R } \right)}}}$. Clearly, when *R* approaches 1, the dissymmetry factor near the nodes of SCL field could be enhanced greatly. Therefore, it appears to have stronger twist optical forces near the nodes of SCL field, which might impart greater chiral bias into asymmetric photo-polymerization reactions. Moreover, due to the closely and orderly packing of BSDA monomers in LB films, chiral transfer and amplification occurred during the polymerization process. Thus obvious dissymmetry enhancement for final PDA films could be achieved by application of SCL.

### Characterization

The UV–Vis spectra were recorded on Shimadzu UV-2550 PC spectrophotometer. The circular dichroism (CD) spectra were measured by using a JASCO CD spectrometer J-810, equipped with a heating cell. The possible effect of linear dichroism of the film was removed by rotating the film plate around the incident light. TEM images were recorded on a JEOL-2000 microscope that operated at 200 kV. The photoresist patterns were recorded by OLYMPUS IX71 confocal microscopy. The XRD measurements were performed using a Rigaku AX-G by using a CuKa (*λ* = 0.154 nm) beam.

## Electronic supplementary material

Supplementary Information

## Data Availability

The authors declare that the all data supporting the findings of this study are available within this article and Supplementary Information files, and also are available from the authors upon reasonable request.
