# Author Correction: Dissymmetry enhancement in enantioselective synthesis of helical polydiacetylene by application of superchiral light

**DOI:** 10.1038/s41467-019-10163-7

**Published:** 2019-05-02

**Authors:** Chenlu He, Guang Yang, Yan Kuai, Sizhen Shan, Lin Yang, Jingang Hu, Douguo Zhang, Qijin Zhang, Gang Zou

**Affiliations:** 1 CAS Key Laboratory of Soft Matter Chemistry, Department of Polymer Science and Engineering, iChEM, University of Science and Technology of China, 230026 Hefei, Anhui China; 2 College of Science, Northeast Forestry University, 150040 Harbin, China; 3 Department of Optics and Optical Engineering, Institute of Photonics, University of Science and Technology of China, Number 96 Jinzhai Road, 230026 Hefei, Anhui China

**Keywords:** Photocatalysis, Polymer characterization, Polymerization mechanisms

Correction to: *Nature Communications* 10.1038/s41467-018-07533-y, published online 30 November 2018.

The original version of the Article contained an error in Fig. 2 in which the TEM images in Fig. 2b and d were incorrect. The correct version of Fig. 2 is:

which replaces the previous incorrect version:

Additionally, the seventh sentence of the ‘Mechanism for the dissymmetry enhancement of SCL field’ section of the Methods originally incorrectly read ‘Since the relative light intensity ratio $R = \frac{{I_2}}{{I_1}}\frac{{E_2^2}}{{E_1^2}}$, the dissymmetry factor near the nodes of SCL field can be expressed as ‘$g_{{\mathrm{SCL}}} = g_{{\mathrm{CPL}}} \times \frac{{\left( {1 + \sqrt R } \right)}}{{\left( {1 - \sqrt R } \right)}}$.’ The correct version states ‘$R = \frac{{I_2}}{{I_1}} = \frac{{E_2^2}}{{E_1^2}}$’ instead of ‘$R = \frac{{I_2}}{{I_1}}\frac{{E_2^2}}{{E_1^2}}$’. This has been corrected in both the PDF and HTML versions of the Article.

