# Supplementary material for: Dissymmetry enhancement in enantioselective synthesis of helical polydiacetylene by application of superchiral light
Source: Nat Commun. 2018 Nov 30;9:5117. doi: 10.1038/s41467-018-07533-y (PMC6269450; doi:10.1038/s41467-018-07533-y)
Supplement: Supplementary file 1 — Supplementary Information [file 41467_2018_7533_MOESM1_ESM.pdf]

Supplementary Information

Dissymmetry enhancement in enantioselective synthesis of helical polydiacetylene by application of superchiral light

He et al.

Table of Contents

Supplementary Fig. 1| The spatial period of SCL field……………………………………………3
Supplementary Fig. 2| The thickness of BSDA films. 4
Supplementary Fig. 3| AFM characterization. 5
Supplementary Fig. 4| UV absorption spectra. 6
Supplementary Fig. 5| CD spectra. 7
Supplementary Fig. 6| UV and CD spectra upon thermal treatment. 8
Supplementary Fig. 7| Anisotropy spectra..9
Supplementary Fig. 8| SHG-LD characterization. 10
Supplementary Fig. 9| Photo-polymerization kinetics data.……………..……………………11
Supplementary Tab. 1| Photo-polymerization kinetic constant and correlation coefficients…11
Supplementary Fig. 10 | Time-resolved development of CD and UV-vis absorbance.……....12
Supplementary Tab. 2 | Different light intensities of two counter-propagating CPL waves…..13
Supplementary Fig. 11 | Asymmetric photo-polymerization experiments. 14
Supplementary Fig. 12 | Molecular structure of chiral compounds. 15
Supplementary Fig. 13 | The CR value of the films polymerized with CPL .…………………16
Supplementary Fig. 14 | Discrimination experiments for PPA enantiomers. …………………17
Supplementary Fig. 15 |Enantioselective discrimination of PEA enantiomers..……………..18
Supplementary Fig.16 | Enantioselective discrimination of PEAM enantiomers.………..…..19
Supplementary Fig. 17 | Enantioselective discrimination of PPAM enantiomers. ……………20

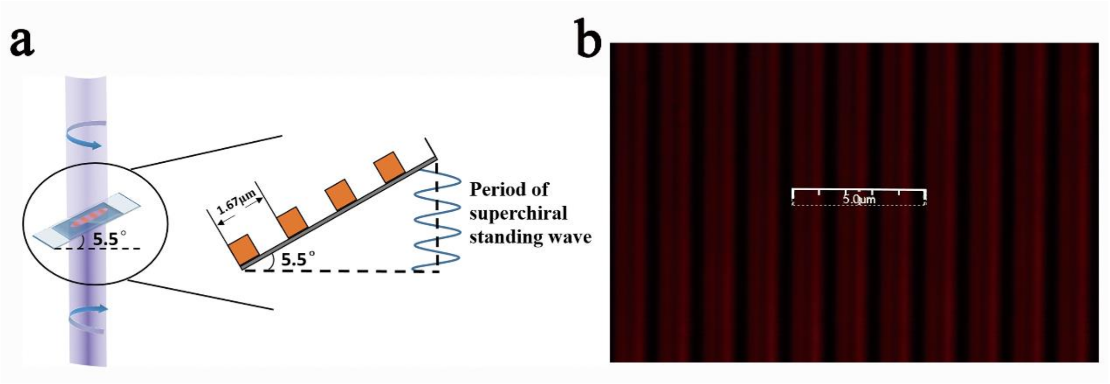

Supplementary Fig. 1| The spatial period of SCL field. **a**, The proposed experiment to probe the spatial period of the SCL field generated by the interference of two counter-propagating CPL beams. **b**, The photoresist patterns recorded by confocal microscopy. The period contained three gratings was recorded about 5.0 μm.

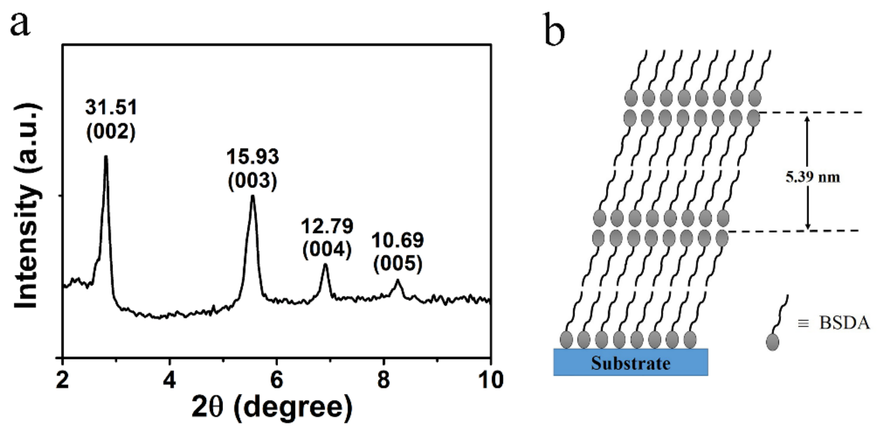

Supplementary Fig. 2| The thickness of BSDA films. a, Small angle X-ray diffraction profile of thus-formed 61-layers BSDA LB films. **b**, Schematic representation of the bilayered architecture obtained from BSDA assemblies, the average spacing of bilayered architecture was 5.39 nm.

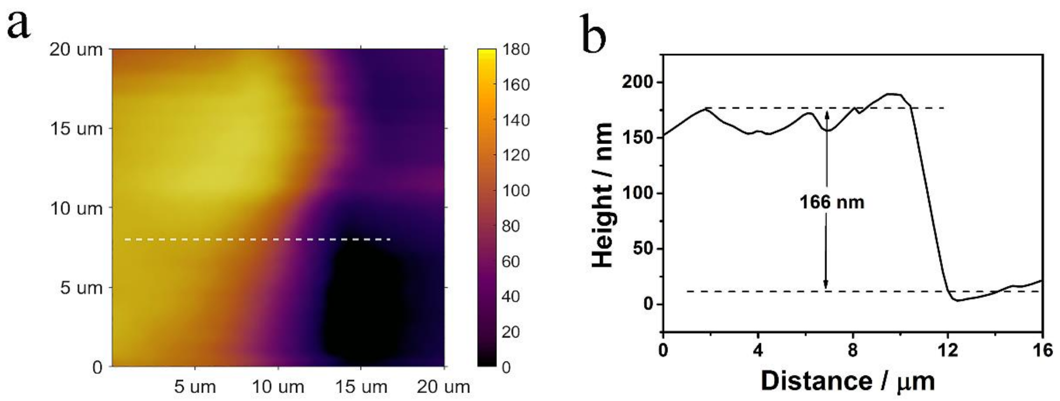

Supplementary Fig. 3| AFM characterization. a, AFM image of BSDA film. **b**, The average thickness of film. The step height was obtained by subtracting the lower cursor position from the upper, giving films thickness of 166 nm.

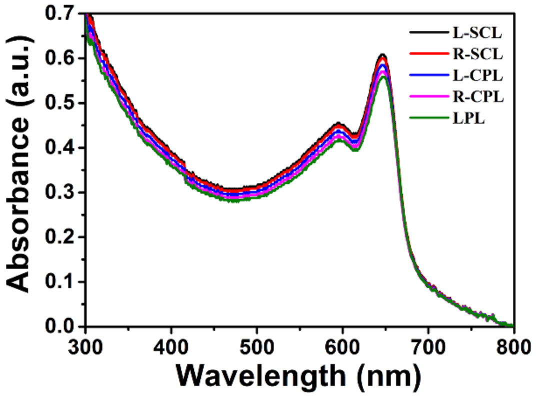

Supplementary Fig. 4| UV absorption spectra. UV-vis absorption spectra of BSDA films upon irradiation with left- and right-handed SCL, CPL or LPL, respectively.

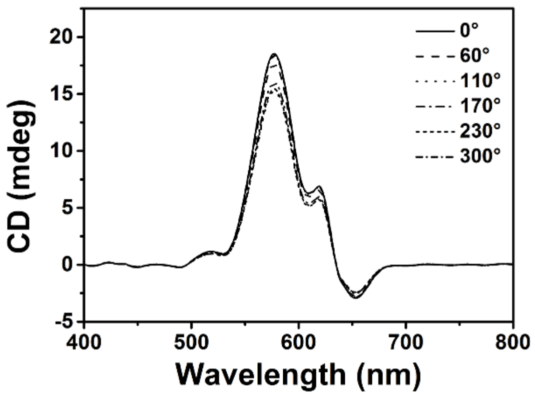

Supplementary Fig. 5| CD spectra. CD spectra at various rotation angles about surface normal for the samples polymerized with left-handed SCL. The signal intensity hardly changed with various rotation angle, indicating that the main origin of CD signals should be the helix formation of PDA chains.

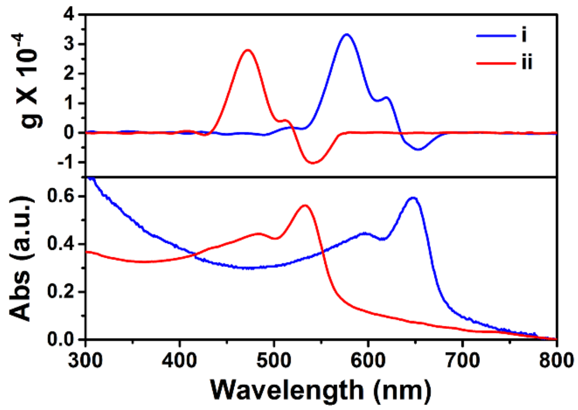

Supplementary Fig. 6| UV and CD spectra upon thermal treatment. UV-vis absorption spectra and CD spectra of chiral PDA films prepared with left-handed SCL: (i) before and (ii) after annealing at 70°C for 10 min.

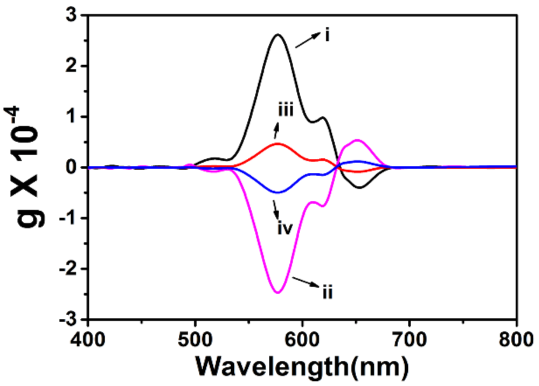

Supplementary Fig. 7| Anisotropy spectra. The g values for chiral PDA films upon irradiation with: (i) left- or (ii) right-handed SCL; (iii) left- or (iv) right-handed CPL, respectively.

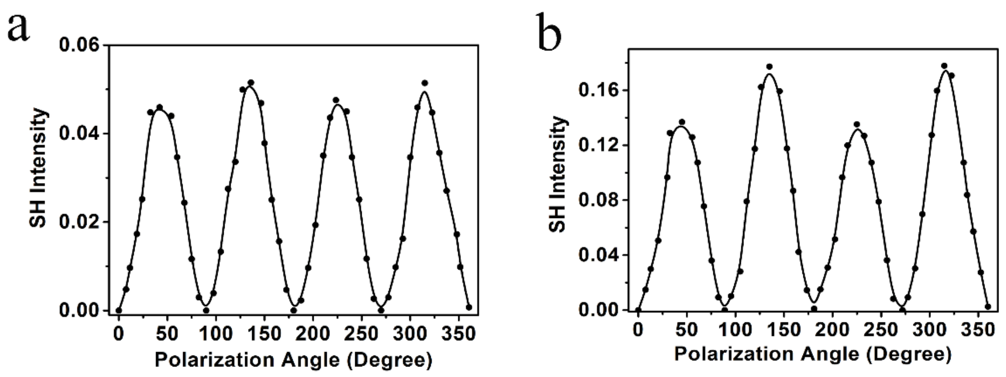

Supplementary Fig. 8| SHG-LD characterization. Polarization dependence curves of SH intensity from chiral PDA films upon irradiation with left-handed **a**, CPL or **b**, SCL. The dots represented the experimental results, and the solid curves were fitting results. All the SH intensity data have been normalized.

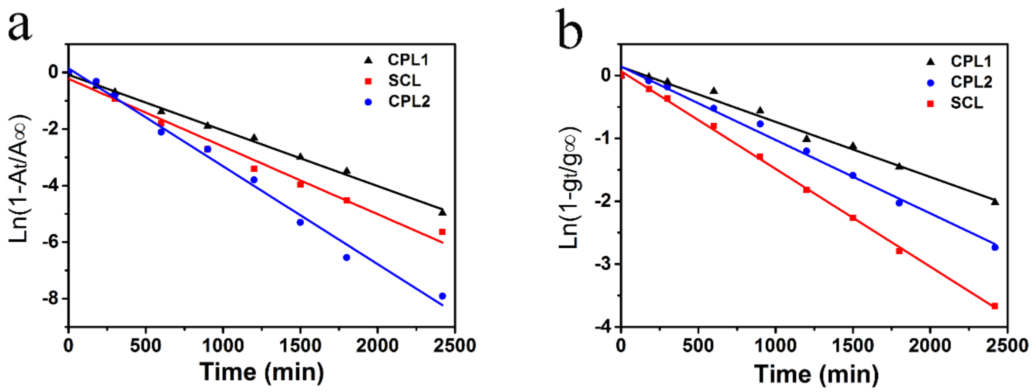

Supplementary Fig. 9| Photo-polymerization kinetics data. a, The polymerization kinetic data obtained from the in situ UV-vis spectra for PDA chains upon irradiation with SCL (red lines), CPL1 (4.0 mW·cm^{-2}, black lines) and CPL2 (6.8 mW·cm^{-2}, blue lines). **b**, The asymmetric polymerization kinetic data obtained from CD spectra for PDA chains upon irradiation with SCL, CPL1 and CPL2. The straight lines were linear regression fits to the corresponding data points.

Supplementary Tab. 1| Photo-polymerization kinetic constant and correlation coefficients for linear regression lines. The photo-polymerization rate constant k, asymmetric photo-polymerization rate constant k' and corresponding R^2 values.

	k (s^{-1})	k' (s^{-1})
CPL1(4.0 mW·cm^{-2})	2.0×10^{-3} (R^2=0.99)	0.8×10^{-3} (R^2=0.98)
SCL	2.7×10^{-3} (R^2=0.99)	1.1×10^{-3} (R^2=0.99)
CPL2(6.8 mW·cm^{-2})	3.1×10^{-3} (R^2=0.98)	1.5×10^{-3} (R^2=0.98)

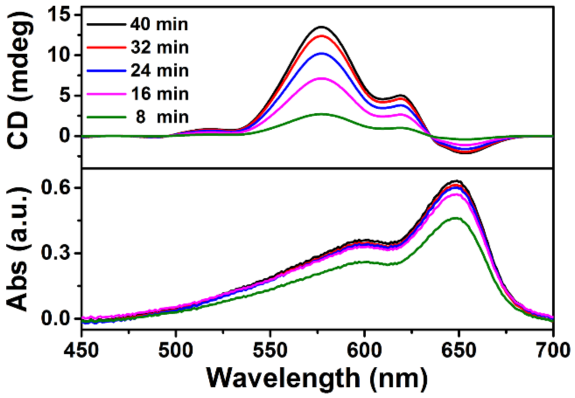

Supplementary Fig. 10| Time-resolved development of CD and UV-vis absorption spectra. The g value extremum at 577 nm increased with the increasing of irradiation time.

Supplementary Tab. 2| The different light intensities of the two counter-propagating CPL waves.

Relative light intensity	Left-handed SCL		Right-handed SCL	
	L- CPL ($mW\cdot cm^{-2}$)	R-CPL ($mW\cdot cm^{-2}$)	L- CPL ($mW\cdot cm^{-2}$)	R-CPL ($mW\cdot cm^{-2}$)
20%	4.0	0.8	0.8	4.0
30%	4.0	1.2	1.2	4.0
40%	4.0	1.6	1.6	4.0
50%	4.0	2.0	2.0	4.0
60%	4.0	2.4	2.4	4.0
70%	4.0	2.8	2.8	4.0
80%	4.0	3.2	3.2	4.0
90%	4.0	3.6	3.6	4.0
95%	4.0	3.8	3.8	4.0

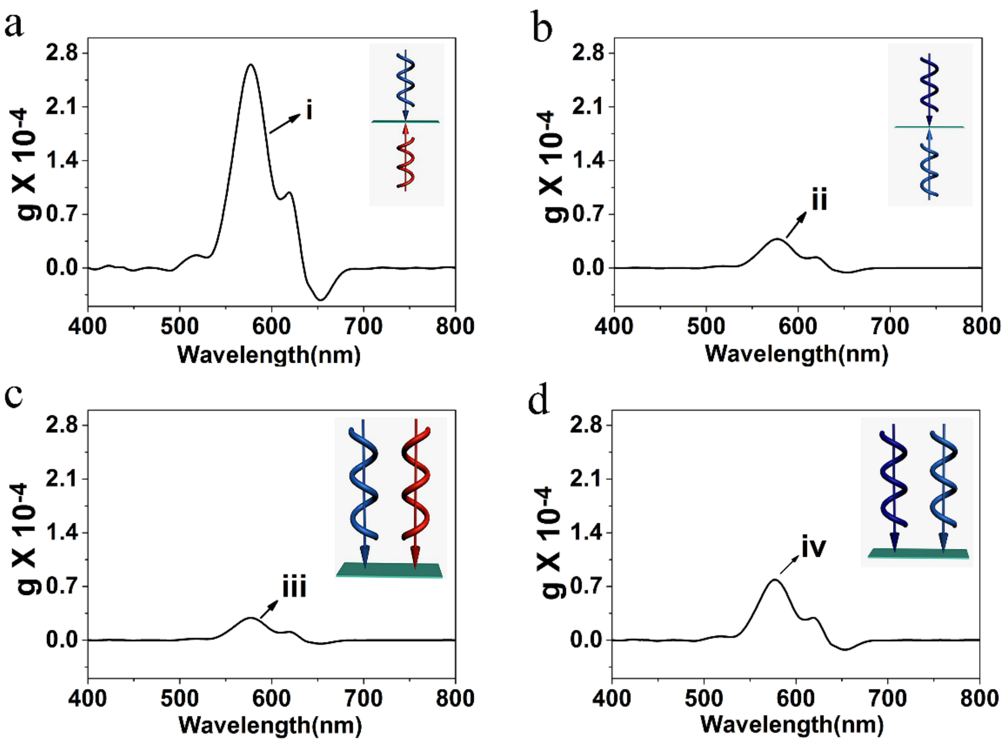

Supplementary Fig. 11| Asymmetric photo-polymerization experiments. The g factor values of final PDA films upon irradiation with two counter-propagating CPL beams with **a**, opposite or **b**, same handedness, or two CPL propagated along same direction with **c**, opposite or **d**, same handedness, respectively. The insert exhibited the intensity of two CPL beams, 4 $mW\cdot cm^{-2}$ (light blue) and 2.8 $mW\cdot cm^{-2}$ (red and dark blue), respectively.

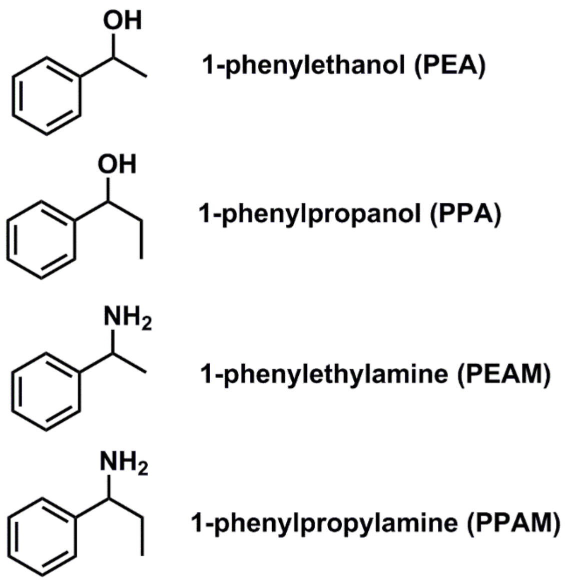

Supplementary Fig. 12| Molecular structure of enantiomers. Molecular structure of PEA, PPA, PEAM and PPAM.

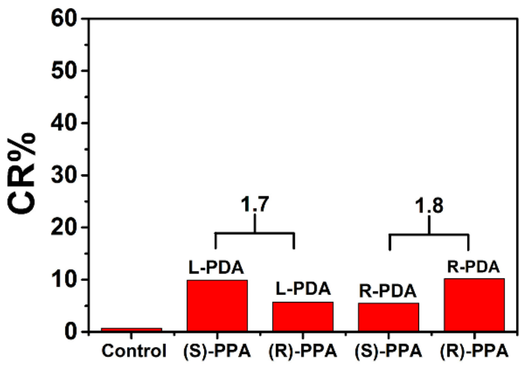

Supplementary Fig. 13| The CR value of the films polymerized with CPL. The CR values of chiral PDA films prepared with conventional CPL on response to R- or S-type PPA solution.

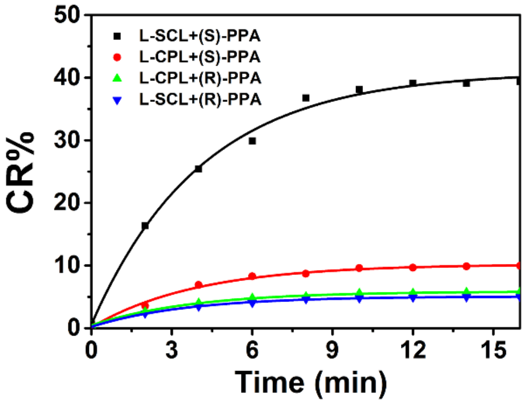

Supplementary Fig. 14| Discrimination experiments for PPA enantiomers. The chronological development of the CR values of PDA films prepared with left-handed SCL or CPL on response to S- or R-type PPA enantiomers, respectively. Solid curves were fitted with single exponential function.

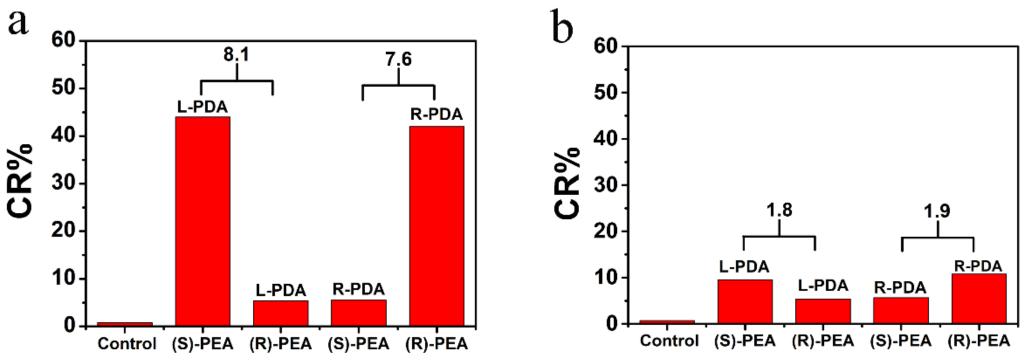

Supplementary Fig. 15|Enantioselective discrimination of 1-phenylethanol enantiomers. The CR value of chiral PDA films prepared with **a,** SCL or **b,** CPL on response to R- or S-type PEA solution, respectively.

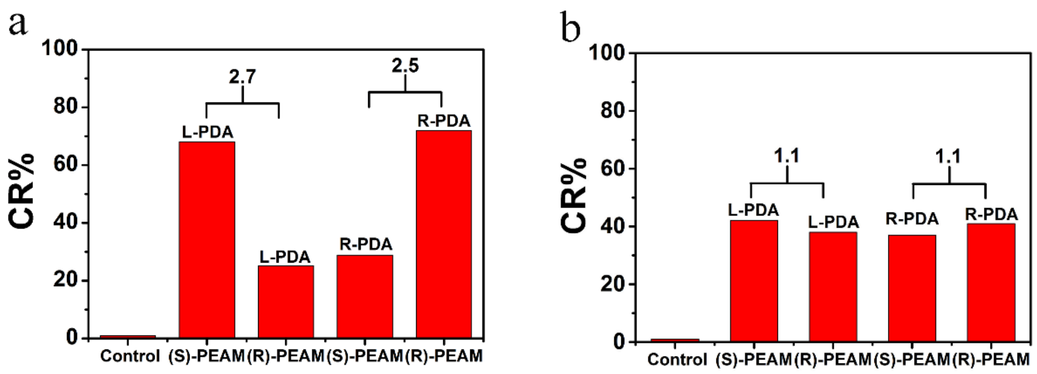

Supplementary Fig. 16|Enantioselective discrimination of 1-phenylethylamine enantiomers. The CR value of chiral PDA films prepared with **a,** SCL or **b,** CPL on response to R- or S-type PEAM solution **(**1×10^{-5} mol·L^{-1}**)**, respectively.

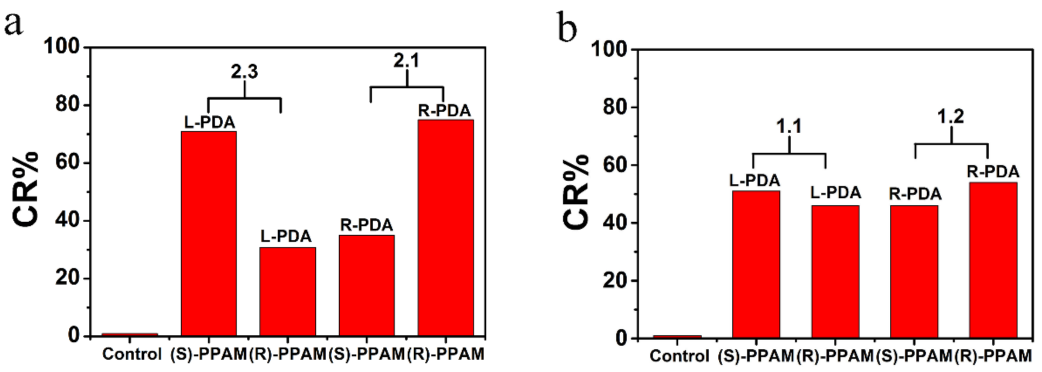

Supplementary Fig. 17|Enantioselective discrimination of 1-phenylpropylamine enantiomers. The CR value of chiral PDA films prepared with **a,** SCL or **b,** CPL on response to R- or S-type PPAM solution **(**1×10^{-5} mol·L^{-1}**)**, respectively.
